# Ameliorative Potential of Vitamin E and Selenium Against Di-2-Ethylhexyl Phthalate (DEHP)–Induced Toxicity in Adult Female Mice

**DOI:** 10.1155/vmi/6844730

**Published:** 2025-11-20

**Authors:** Md. Samiul Haque, Md. Hosne Mobarak, Md. Khayrul Basher, Sumon Sarkar, Sourav Sarker, Md. Rashedul Islam

**Affiliations:** ^1^Department of Genetics and Animal Breeding, Hajee Mohammad Danesh Science and Technology University, Dinajpur 5200, Bangladesh; ^2^Department of Physiology and Pharmacology, Hajee Mohammad Danesh Science and Technology University, Dinajpur 5200, Bangladesh; ^3^Green Life Medical College, MAK Khan Tower 30, Bir Uttam K. M. Shafiullah Sarak, Green Road, Dhaka 1205, Bangladesh

**Keywords:** female mice, hematological and biochemical parameters, organ-to-body weight ratio, phthalates, sodium selenite, vitamin E

## Abstract

Di-2-ethylhexyl phthalate (DEHP) is an omnipresent environmental toxicant with significant potential for human exposure. It primarily escapes from plastic packaging used for food and water. DEHP exposure has been linked to several health hazards, including cancer, cardiovascular diseases, and organ toxicity, disrupting the endocrine system as well as affecting biological processes. The present study investigates the protective effects of Vitamin E and selenium against DEHP-induced toxicity in adult female mice. In this study, adult female mice (Swiss Albino) were randomly categorized into five groups: control, DEHP, DEHP + Vitamin E, DEHP + Na_2_SeO_3_, and DEHP + Vitamin E + Na_2_SeO_3_. From Day 49 to Day 61 of the treatment period, the animals were administered orally 600 mg/kg body weight of DEHP, 200 mg/kg body weight of Vitamin E, and 1 mg/kg body weight of Na_2_SeO_3_. After treatment, body weight, organ-to-body weight ratio, and hematological and biochemical parameters were assessed. DEHP exposure caused a significant decrease in final body weight, body weight gain, and rate of body weight gain, but DEHP + Vitamin E, DEHP + Na_2_SeO_3_, and DEHP + Vitamin E + Na_2_SeO_3_ groups lessened it. While considering blood parameters, the group exposed to DEHP showed a notable rise in white blood cells (WBCs). Furthermore, the DEHP group significantly increased random blood sugar (RBS), serum glutamic oxaloacetic transaminase (SGOT), and alkaline phosphatase serum levels. Nevertheless, these levels were notably decreased in the groups who received treatment with Vitamin E and sodium selenite (Na_2_SeO_3_). Phthalate exposure also led to a significant increase in the organ-to-body weight ratio in the spleen and slight discoloration with necrotic foci present in the liver compared to the control group. It is remarkable that Vitamin E and Na_2_SeO_3_ separately or synergistically mitigated all the changes. The present investigation provides evidence that Vitamin E and sodium selenite can minimize phthalate-induced damage in adult female mice.

## 1. Introduction

Di-2-ethylhexyl phthalate (DEHP) is a well-known phthalate frequently used as a plasticizer to improve the adaptability and use of polymers. In Bangladesh, the plastic manufacturing sector is expanding at a pace of 20% on average per year [1]. Two million tons of DEHP are produced annually around the world [2]. It is extensively utilized in many sectors, such as textiles, wires and tubes, vinyl gloves, wall and floor paints, toys, and medical equipment, and chemicals often flow from detergents and fragile oils for paints and adhesives. That weak relationship makes it possible for the DEHP to leak or disperse from polymers into food, air, and body fluids, presenting both the environment and humans [3–5]. The most common routes of exposure to DEHP are oral and dermal. The gastrointestinal absorption of phthalates also appears to be high; DEHP absorption is more than 50%. In addition, large numbers of reports emerging in Europe and Asia show much higher levels of phthalate metabolites in the urine, blood, and semen of the plastic industry workers than in the general population [4, 6, 7]. Exposure to DEHP has been associated with gynecological problems, obesity, male infertility, and endocrine disturbance [8–12]. Additionally, it is linked to an increased risk of respiratory illnesses, attention deficits, and unfavorable reproductive consequences [13–16]. Furthermore, research conducted on animals has shown that DEHP is carcinogenic and has a substantial toxic impact on several organs, including the liver, spleen, kidneys, and testes [17, 18]. The precise process by which DEHP is hazardous is still unclear. Nonetheless, a growing amount of data indicate that oxidative stress is a major factor in the toxicities caused by DEHP. Peroxisome proliferator–activated receptor γ (PPARγ) is activated by DEHP, as demonstrated by *in vitro* and *in vivo* investigations, which leads to the production of oxidative stress and disruption of endocrine signaling [12, 19]. Changes in the cellular redox balance in the rat liver were an indicator of the oxidative stress induced by DEHP. According to studies from both humans and animals, DEHP causes cancer through a variety of biochemical pathways, including DNA damage [20, 21]. Consequently, antioxidant modulation using selenium and Vitamin E has been a major focus of this study into pharmaceutical treatments against DEHP-induced toxicity.

Selenium is a vital antioxidant with potential chemoprotective and cancer-preventive properties. It prevents the development of cancer by acting as an antimutagenic agent in healthy cells. The primary cause of these protective actions is its involvement in selenium-containing enzymes (selenoproteins), which guard against oxidative damage to DNA and other cellular constituents [22]. Selenium has been shown in studies on animals to have protective and therapeutic effects, such as reducing inflammation and oxidative stress [23, 24]. The pharmacological effects of selenium-containing compounds include hepatoprotective, cardioprotective, anti-inflammatory, and anticancer properties [25]. Previous studies reported *in vitro* and animal research that Se increases the antioxidant activity to defend against a variety of toxic agents [26].

Moreover, the family of lipid-soluble vitamins known as Vitamin E includes α-tocopherol, which is the most active form and a potent biological antioxidant. In biological systems, Vitamin E may successfully reduce oxidative stress, LPO, and the harmful consequences of ROS [27]. There is also evidence supporting the anti-inflammatory, antiplatelet aggregation, and immune-boosting qualities of α-tocopherol [28]. These characteristics make α-tocopherol thought to offer protection against a wide range of illnesses, such as cancer, cardiovascular, neurological, and reproductive conditions [29–32]. According to recent investigations, α-tocopherol has an antioxidative impact against some environmental toxicants [33, 34].

Co-exposure to Vitamin E and selenium (Se), together with environmental toxicants, considerably decreases oxidative stress because of their numerous antioxidant activities. On the other hand, nothing is known about how co-exposure to Vitamin E and selenium affects toxicity caused by DEHP. This is the first research to assess the protective effects of Vitamin E and selenium on DEHP-induced changes in hematological and biochemical profiles, impairment of body development, and toxicity to internal organs in adult female mice.

## 2. Materials and Methods

### 2.1. Animal Care and Housing

Laboratory-bred Swiss albino female mice, 6–8 weeks old and weighing 25–30 g, were obtained under the suggested guidelines from the animal resources facility of the International Center for Diarrheal Disease Research, Bangladesh ((ICDDR, B) at the laboratory animal research facilities of the Department of Genetics and Animal Breeding, Hajee Mohammad Danesh Science and Technology University (HSTU), Dinajpur. They were housed in an animal building that had plenty of ventilation. The habitat allowed the animals complete access to food and water and was maintained at a temperature of 26 ± 2°C with a relative humidity of 26%–30%. A 12-h light/dark cycle was adapted for the animals' housing. In plastic cages with wood-cube bedding, five mice were chosen randomly and placed in each cage, and clean, comfortable bedding for the cage was provided. Every morning, drinking water and bedding (made of wood cob) were changed to create a tranquil atmosphere. Regular food was given to them in the form of formulated feed. Water and food were consistently accessible. The quantity of water consumed each day was noted. Different animal groups had been separated from one another. Before starting the study, the mice were kept for a week to adjust to their new environment, and the cages were labeled with the proper introduction. The HSTU Laboratory Animal Care and Use Committee approved all animal studies, which were carried out following the regulations for the care and use of laboratory animals.

### 2.2. Experimental Strategy

Each experimental group consisted of five mice (*n* = 5). The entire experiment was conducted in three independent replicates (*N* = 3), resulting in a total of 15 mice per treatment group (Table 1). DEHP dissolved with the same volume of DMSO solution at a dose of 600 mg/kg body weight, and Vitamin E also dissolved with an equal volume of DMSO solution at a dose of 200 mg/kg body weight. Na_2_SeO_3_ dissolved with distilled water at 1 mg/kg body weight was treated orally using a mouse feed needle (Table 1).

### 2.3. Body Growth of Mice

The body weight of each mouse was measured at 3-day intervals during exposure and after exposure from 49 days to 61 days. After 49–61 days, phthalates, Vitamin E, and sodium selenite was administered, and after 62 days–69 days, the observation weight was also measured by using an electric balance. The weight gain and rate of weight gain for each mouse were calculated and compared between the control, DEHP, DEHP + Vitamin E, DEHP + Na_2_SeO_3_, and DEHP + Na_2_SeO_3_ + Vitamin E; These data were calculated by using the following formula:

(1) $$\begin{array}{c} \text{body weight gain}=\text{final body weight}-\text{initial body weight},\\ \text{rate of body weight gain}=\frac{\text{final body weight}-\text{initial body weight}}{\text{time}}. \end{array}$$

### 2.4. Collection of Blood and Internal Organs

After 69 days of age, mice were starved overnight and given mild diethyl ether anesthesia the next morning to draw blood. Due to the need for a significant volume of blood, a cardiac puncture was used to collect the blood. Blood was then preserved in a collecting tube for hematological and biochemical examination; several organs, including the liver, lung, kidney, spleen, heart, and uterus, were carefully removed for the evaluation of the organ morphology and organ-to-body weight ratio.

#### 2.4.1. Hematological Analysis

Blood samples were collected into lithium heparinized tubes (BD 36666; Thermo Fisher, Waltham, Massachusetts, USA). Proper mixing with the anticoagulant was ensured immediately after collection. Then, every sample was sent to the laboratory for the measurement of hematological parameters such as complete blood count (CBC), including red blood cells (RBCs), white blood cells (WBCs), different blood counts (neutrophils, eosinophils, basophils, monocytes, and lymphocytes), platelets, hemoglobin, packed cell volume, erythrocytes sedimentation rate, trichloroethylene, mean capsular volume (MCV), mean hemoglobin concentration (MCHC), mean corpuscular hemoglobin (MCH), a standard deviation of RBC distribution width (RDW-SD), and coefficient of variation of RDW (RDW-CV), which were determined using an automated hematology analyzer (BC-20; Mindray, Shenzhen, China) and compared among groups.

#### 2.4.2. Biochemical Evaluation

Immediately after being drawn, blood samples were placed into clot activator tubes. The coagulation process was triggered via a clot activator tube with silica particles on the inner walls. Each sample was given a minimum of 30 min and a maximum of 60 min to clot. After clotting, the sample was centrifuged at 600 rpm for 10 min, and then, serum or plasma was pipetted from the cellular elements (erythrocytes, platelets, leukocytes) and transferred to an acid-washed polypropylene tube, properly labeled, and analyzed. Random blood sugar (RBS), serum creatinine, total cholesterol, triglyceride, uric acid, serum glutamic oxaloacetic transaminase (SGOT), serum glutamine phosphatase transaminase (SGPT), and alkaline phosphatase. A biochemical analyzer (18200; HUMAN, Wiesbaden, Germany) and commercially available reagent (RANDOX, Crumlin, County Antrim, United Kingdom) were used.

#### 2.4.3. Evaluation of Organs

##### 2.4.3.1. Organ Morphology and Organ-to-Body Weight Ratio

After collecting, the organs were washed with distilled water to remove blood. As a result, the organ was indisputably visualized to evaluate gross morphology (position, shape, size, and consistency). Organ size was determined using a centimeter scale, and weight was determined by a digital weight machine. The organ-to-body weight ratio was calculated by the following formula: organ-to-body weight ratio = (Organ weight/Bodyweight) × 100. Then, it was compared among groups.

### 2.5. Statistical Analysis

Each treatment group consisted of five mice (*n* = 5), and the entire experiment was independently repeated three times (*N* = 3), giving a total of 15 animals per group. Data are presented as mean ± SEM. Statistical analyses were performed using one-way analysis of variance (ANOVA), followed by post hoc multiple comparison tests to determine the differences among groups. Variability across replicates was incorporated into the calculation of the SEM. A two-sided *p* value < 0.05 was considered statistically significant. All analyses were conducted using SPSS software (Version 16; SPSS Inc., Chicago, IL, USA).

## 3. Results

### 3.1. Protective Potential of Vitamin E and Selenium Against the Toxic Effects of Phthalates on the Body Growth of Mice

#### 3.1.1. Body Growth Alterations Were Found Among Treatment Groups

**Figure 1:**
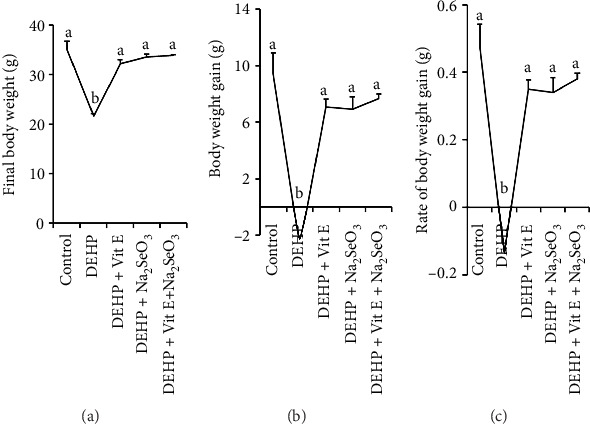
Protective effect of Vitamin E and selenium against the toxic effects of phthalate on the body growth of adult female mice. The effect of phthalate, Vitamin E, and selenium on body growth in control and treated mice was assessed and compared according to final body weight (a), body weight gain (b), and rate of body weight gain (c). Values are presented as mean ± SEM of the three replicates. Significant difference is indicated by different letters, a and b.

This research outcome showed that final body weight (Figure 1(a)), body weight gain (Figure 1(b)), and body weight gain (Figure 1(c)) due to DEHP exposure were significantly reduced compared to the control, DEHP + Vitamin E, and DEHP + Vitamin E + Na_2_SeO_3_ groups. But interestingly, exposure to Vitamin E, Na_2_SeO_3_, and Vitamin E with Na_2_SeO_3_ at the same concentration as the DEHP group significantly increased the final body weight (Figure 1(a)), body weight gain (Figure 1(b)), and rate of body weight gain (Figure 1(c)) compared to the DEHP group.

### 3.2. Vitamin E and Selenium Ameliorate the Hematological Parameters Induced by Phthalate

**Figure 2:**
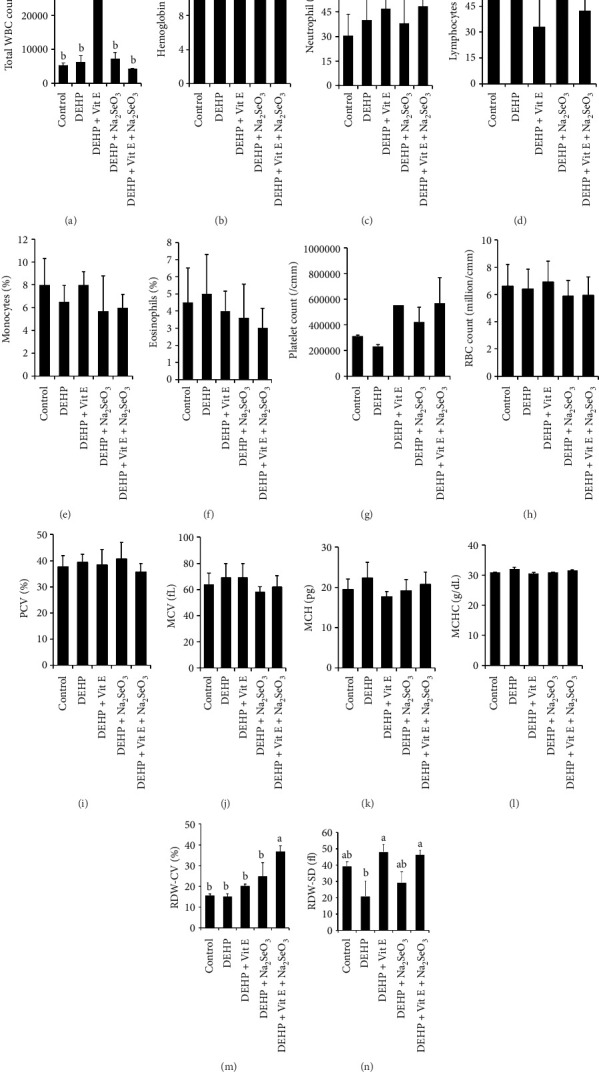
Effects of Vitamin E and selenium on hematological changes induced by phthalate hematological parameters, namely, total WBC count, hemoglobin (g/dL), neutrophil (%), lymphocytes (%), monocyte (%), eosinophils (%), platelet count (/cmm), RBC count (million/cmm), PCV (%), MCV (fL), MCH (pg), MCHC (g/dL), RDW-CV (%), and RDW-SD (fL) (a–n) of control with DEHP, DEHP + Vitamin E, DEHP + Na_2_SeO_3_, and DEHP + Vitamin E + Na_2_SeO_3_ were assessed and compared. Data are presented as mean ± SEM (*n* = 3). Statistical significance is indicated by letter (a, b). MCH = mean corpuscular hemoglobin, MCHC = mean corpuscular hemoglobin concentration, MCV = mean corpuscular volume, PVC = packed cell volume, RBC = red blood cell, RDW-CV = coefficient of variation of red blood cell distribution width, RDW-SD = standard deviation of red blood cell dimension width, TC = total count, and WBC = white blood cell.

The present study was aimed at measuring the changes in the hematological parameters of blood tissue compared among control, DEHP-, DEHP + Vitamin E–, DEHP + Na_2_SeO_3_–, and DEHP + Vitamin E + Na_2_SeO_3_–exposed groups (Figure 2) in adult female mice. The hematological test revealed a significant increase in the WBC count of the DEHP + Vitamin E group compared to the DEHP group (Figure 2(a)). The RDW-CV of the DEHP + Vitamin E + Na_2_SeO_3_ group is significantly higher than that of the control, DEHP, DEHP + Vitamin E, and DEHP + Na_2_SeO_3_ groups (Figure 2(m)). The DEHP group had considerably lower RDW-SD levels in comparison with the DEHP + Vitamin E and DEHP + Vitamin E + Na_2_SeO_3_ groups (Figure 2(n)).

### 3.3. Vitamin E and Selenium Improve the Changes in Biochemical Parameters of Phthalate-Induced Toxicity

**Figure 3:**
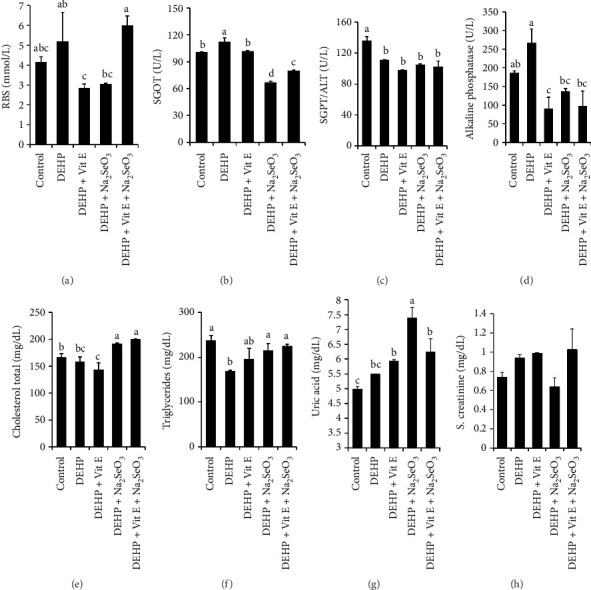
Vitamin E and selenium improve the serum biochemical alteration against phthalate-induced toxicity. Biochemical parameters such as RBS (mg/dL), SGOT (U/L), SGPT (U/L), alkaline phosphatase (U/L), total cholesterol (mg/dL), triglyceride (mg/dL), uric acid (mg/dL), and serum creatinine (mg/dL) (a–h) are assessed and compared among control, DEHP-, DEHP + Vitamin E–, DEHP + Na_2_SeO_3_–, and DEHP + Vitamin E + Na_2_SeO_3_–exposed groups. Values are presented as mean ± SEM (*n* = 3). Statistical significance is indicated by letters (a, b, c, d). RBS = random blood sugar, SGOT = serum glutamic oxaloacetic transaminase, and SGPT = serum glutamine phosphatase transaminase.

The results of the study's serum biochemical examination showed that the RBS level of the DEHP + Vitamin E group was substantially lower than that of the DEHP and DEHP + Vitamin E + Na_2_SeO_3_ groups (Figure 3(a)). Additionally, there was a significant increase in the SGOT level of the DEHP group compared among control, DEHP + Vitamin E–, DEHP + Na_2_SeO_3_–, and DEHP + Na_2_SeO_3_ + Vitamin E–exposed groups. Interestingly, when sodium selenite and Vitamin E were exposed, this value was significantly lower than the DEHP groups (Figure 3(b)). In the case of SGPT, the control group is remarkably higher than other groups (Figure 3(c)). The alkaline phosphatase level is notably increased in the DEHP group, but when we induced Vitamin E or Na_2_SeO_3_ or a combination, this level outstandingly fell off (Figure 3(d)). Cholesterol levels in the DEHP + Vitamin E + Na_2_SeO_3_ and DEHP + Na_2_SeO_3_ groups are more enriched than those in the control, DEHP, and DEHP + Vitamin E groups (Figure 3(e)). In DEHP, the triglyceride level is particularly minimal compared to the control, DEHP + Na_2_SeO_3_, and DEHP + Vitamin E + Na_2_SeO_3_ groups (Figure 3(f)). In this study, when we compare the uric acid levels in different treatment groups, this value is particularly enriched in the DEHP + Na_2_SeO_3_ group compared to the control, DEHP, DEHP + Vitamin E, and DEHP + Na_2_SeO_3_ groups (Figure 3(g)).

### 3.4. Organ-to-Body Weight Ratio Evaluation

Vitamin E and selenium's ability to restore phthalate-induced toxicity was evaluated by measuring body weight and the organ-to-body weight ratio. Adult female mice were used in this investigation, and the relative weights of the liver, lung, kidney, spleen, heart, uterus, and urine bladder, as well as the organ-to-body weight ratio, were measured and compared between the exposed groups and the control group. The outcome demonstrated several notable organ-specific variations between the control group and treatment groups. When exposed to DEHP, the liver weight increased considerably compared to the control group, but it dropped significantly in the DEHP + Vitamin E, DEHP + Na_2_SeO_3_, and DEHP + Vitamin E + Na_2_SeO_3_ groups (Table 2). The heart weight ratio notably rose in the DEHP + Vitamin E + Na_2_SeO_3_ group when we compared it to the other treatment groups. Spleen weight is higher in the DEHP group, which differs from the normal level (Table 2).

### 3.5. The Protective Action of Vitamin E and Selenium Against the Toxicity of Organ Morphology Caused by Phthalates

**Figure 4:**
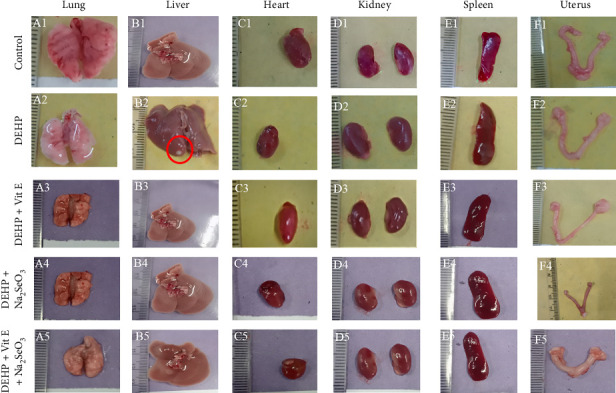
Protective effect of Vitamin E and selenium on phthalate-induced morphological alteration of the liver, lung, heart, kidney, spleen, and uterus. After the sacrifice of mice, the internal organs, namely, lung (A1, A2, A3, A4, A5), liver (B1, B2, B3, B4, B5), heart (C1, C2, C3, C4, C5), kidney (D1, D2, D3, D4, D5), spleen (E1, E2, E3, E4, E5), and uterus (F1, F2, F3, F4, F5), were collected, photographed, and compared among control, DEHP-, DEHP + Vitamin E–, DEHP + Na_2_SeO_3_–, and DEHP + Vitamin E + Na_2_SeO_3_–exposed group of adult female mice.

Visual observation of morphological characteristics of different organs showed slight discoloration of the liver, which was subsequently affected in the phthalate-treated mouse compared to the control group and different treatment groups (Figure 4 B2, B1, B3, B4, B5). Interestingly, the size of the liver in the phthalates group (Figure 4 B2) was larger than in other groups (Figure 4 B1, B3, B4, B5). On the contrary, normal morphological features of the kidney, lung, liver, spleen, heart, uterus, and urinary bladder were observed in control, DEHP-, DEHP + Vitamin E, DEHP + Na_2_SeO_3_–, DEHP + Vitamin E + Na_2_SeO_3_–treated adult female mice (Figure 4).

## 4. Discussion

Human health relies on the essential presence of dietary antioxidants. There is increasing evidence that the toxicity of DEHP is linked to oxidative stress and the reduction of antioxidants in many tissues, including the spleen [35]. Consequently, the supplementation of antioxidants might slow down the oxidative damage and negative consequences of DEHP exposure. Vitamin E and selenium are essential antioxidants that mitigate the harmful effects of phthalate-induced oxidative stress, hematological alterations, and toxicity to reproductive and vital organs [18, 36]. Research has demonstrated that the combination of Vitamin E, and selenium has a synergistic impact in providing protection [37]. The current study investigated the impact of concurrent exposure to Vitamin E, selenium, and DEHP on the hematological and biochemical profiles of blood, impaired body growth, and damage to internal organs in adult female mice. Our study showed that the final body weight, body weight gain, and rate of body weight gain were significantly lower in the DEHP group than in the control group. This result is consistent with earlier study findings noted by Aydemir et al. [38–40]. Phthalates can interfere with the functioning of the gastrointestinal system, causing problems with the absorption and use of nutrients. This directly affects the process of development. These compounds have the potential to harm the protective lining of the digestive system and disrupt the community of micro-organisms living in the gut, resulting in inflammation and impaired absorption of nutrients, which can impede normal growth processes [41, 42]. However, Vitamin E and Se supplementation improves body growth in the DEHP + Vitamin E, DEHP + Na_2_SeO_3_, and DEHP + Vitamin E + Na_2_SeO_3_ groups. Vitamin E and selenium are both potent antioxidants. Vitamin E acts as an antioxidant, counteracting free radicals and so avoiding oxidative stress and cellular harm. Selenium is a constituent of glutathione peroxidase, an enzyme that safeguards cells against oxidative harm. Selenium supplementation increases glutathione peroxidase activity and interacts with inflammatory indicators to have a positive effect and enhance the function of Vitamin E. This combination can aid in diminishing the oxidative stress induced by toxic substances [43–46]. According to Huang et al., the improvement in body weight is caused by the positive effects of selenium and Vitamin E on the gut flora. It has been suggested that there is a synergistic relationship between selenium and Vitamin E because GSHPx continues the work of Vitamin E by detoxifying hydroperoxides. Females exposed to phthalates showed a substantial increase in liver weight when compared to the control group. Studies have shown a considerable rise in liver weight that is consistent with the findings of Aydemir et al. [38, 47–49]. According to Pugh et al., this modification and the suppression of GJIC are thought to have a role in the development of mouse liver cancer [50]. Hypertrophy (an increase in the size of each cell) and hyperplasia (an increase in the number of cells) of liver parenchymal cells appear to be causes of phthalate exposure to rats. Notably, the administration of Vitamin E and selenium, both separately and together, resulted in a considerable decrease in liver weight after exposure to phthalates. Prior research has demonstrated that selenium and Vitamin E enhance antioxidant levels and offer significant safeguarding to the liver against toxicity through their antioxidant and free radical-neutralizing properties [51–53]. According to Fan et al., the spleen is a good indicator of an organism's development and immunological function [54]. The study revealed a substantial rise in spleen weight in the DEHP group as compared to the control group. This is corroborated by prior research that suggests DEHP triggers oxidative stress in the spleen by disrupting the Nrf2 signaling pathway and modifying the transcription of its downstream genes. Significantly, the examination of quail spleen tissue subjected to DEHP showed an expansion of the splenic corpuscular boundary and an increase in the cell gap [54]. However, Vitamin E and selenium supplementation showed no significant changes in spleen weight compared to the DEHP group of exposure. Hematological and biochemical characteristics serve as dependable markers for evaluating the state of health in both people and animals [55]. The amount of WBCs, which is an important indicator of the body's inflammatory response, might vary depending on the type of treatment administered. The group treated with DEHP plus Vitamin E (DEHP + Vitamin E group) has a significantly elevated WBC count in comparison with both the control group and the DEHP group. Conversely, the DEHP group alone displays a reduced WBC count. Decreased WBC counts are linked to immunological dysfunctions, including autoimmune illnesses, immune malignancies, infections, and bone marrow problems [56]. On the other hand, Vitamin E counteracts these effects by diminishing the generation of free radicals, lowering the synthesis of PGE2, and augmenting the creation of IL-2, which regulates immunological responses [57, 58].

A substantial increase in blood RBS, SGOT, and alkaline phosphatase levels was seen in the DEHP-exposed group compared to the control group. These findings are consistent with prior research conducted by Baralić et al. [59–61], but in the case of SGPT, the level is opposed to previous findings. Phthalate exposure has been demonstrated to decrease pancreatic insulin production, trigger beta-cell demise, and modify their ultrastructure. Moreover, there is evidence linking DEHP exposure and DNA damage in both people and rodents [62]. Direct impacts on beta cells, resulting in decreased insulin production, have also been shown in previous research [63]. Heightened levels of SGOT and alkaline phosphatase, crucial liver enzymes, suggest liver injury, as these enzymes are secreted into the circulatory system when liver cells are impaired. Notably, both Vitamin E and selenium, whether given together or separately, substantially decrease levels of RBS, SGOT, and alkaline phosphatase in comparison with exposure to DEHP. Consistent with prior research demonstrating that Vitamin E and selenium work together to decrease sodium azide-induced liver toxicity [64] and alleviate stress-induced liver damage [65], this discovery supports the present results. Moreover, the reduction of oxidative damage generated by these antioxidants may contribute to the preservation of pancreatic beta-cell function, thereby enhancing the generation and release of insulin [66]. A visual assessment of the internal organ morphology showed slight changes in color and areas of tissue death in the liver of mice treated with phthalates. The liver seemed notably bigger compared to the other groups. The increase in size is caused by the excessive growth and expansion of liver parenchymal cells caused by DEHP, resulting in higher levels of reactive oxygen species and oxidative DNA damage [17]. The antioxidant characteristics of Vitamin E and selenium counteract oxidative damage and enhance liver function [65]. The kidneys, lungs, liver, spleen, heart, uterus, and urine bladder of control, DEHP-, DEHP + Vitamin E–, DEHP + Na_2_SeO_3_–, and DEHP + Vitamin E + Na_2_SeO_3_–exposed groups exhibited normal morphological features.

From a veterinary clinical perspective, these findings hold practical significance for animal health management. Phthalate exposure also threatens livestock and companion animals through contaminated feed, water, or plastic equipment used in animal production and veterinary care. The observed oxidative and hepatic alterations in mice likely mirror mechanisms that could impair growth, immunity, and reproduction in domestic species. Therefore, the demonstrated protective effects of Vitamin E and selenium supplementation have direct translational relevance for veterinary medicine. Both antioxidants are already incorporated into livestock and pet diets, and the present study supports their broader use as prophylactic or therapeutic agents against environmental toxicants such as DEHP. Integrating antioxidant therapy into herd health and companion-animal care protocols may help reduce oxidative-stress–related organ damage, enhance immune resilience, and improve overall productivity and reproductive efficiency in animals.

## 5. Conclusion

The present study provides evidence supporting the positive impacts of Vitamin E and sodium selenite on several aspects of adult female mice, including body development, organ-to-body weight ratios, and organ morphology, as well as hematological and biochemical markers. Oxidative stress plays a major role in the toxicity of DEHP, and the antioxidant characteristics of Vitamin E and sodium selenite seem to alleviate these impacts. The inclusion of diverse selenoproteins with antioxidants and detoxifying properties might augment defense against damage caused by DEHP. Synergistic effects of Vitamin E and selenium have been detected; however, the specific pathways are yet uncertain. A detailed investigation is required to clarify the molecular processes responsible for the protective actions of these antioxidants against DEHP toxicity. Potential future research should encompass the examination of the transgenerational consequences of DEHP and its improvement, the molecular investigation of oxidative stress indicators (RNA and protein) in mice exposed to DEHP, and the evaluation of the effects of DEHP exposure at relevant doses to humans and also histopathological confirmation of organ lesions, especially liver and spleen.

## Figures and Tables

**Table 1:** Treatment protocol of the experimental groups.

Group	Treatments
1. Control	No treatment
2. DEHP	600 mg/kg body weight/day
3. DEHP + Vitamin E	As in Group 2 and 200 mg vitamin E/kg body weight/day
4. DEHP + Na_2_SeO_3_	As in Group 2 and 1 mg Na_2_SeO_3_/kg body weight/day
5. DEHP + Vitamin E + Na_2_SeO_3_	As in Group 3 and 1 mg Na_2_SeO_3_/kg body weight/day

Abbreviation: DEPH = Di-2-ethylhexyl phthalate.

**Table 2:** Organ-to-body weight ratio of different treatment groups.

Parameters	Treatments				
	Control	DEHP	DEHP + Vitamin E	DEHP + Na_2_SeO_3_	DEHP + Vitamin E + Na_2_SeO_3_
Liver	4.36 ± 0.14^b^	6.75 ± 0.65^a^	4.41 ± 0.31^b^	4.78 ± 0.36^b^	5.01 ± 0.24^b^
Lung	0.59 ± 0.03	0.71 ± 0.03	0.60 ± 0.12	0.84 ± 0.12	0.78 ± 0.06
Kidney	1.03 ± 0.02	1.16 ± 0.06	1.09 ± 0.03	1.15 ± 0.15	1.10 ± 0.01
Heart	0.39 ± 0.01^b^	0.40 ± 0.01^b^	0.41 ± 0.02^b^	0.36 ± 0.02^b^	0.52 ± 0.03^a^
Spleen	0.38 ± 0.04^c^	0.56 ± 0.05^ab^	0.58 ± 0.01^b^	0.43 ± 0.04^b^	0.55 ± 0.03^b^
Urinary bladder	0.03 ± 0.01	0.05 ± 0.01	0.03 ± 0.01	0.05 ± 0.01	0.05 ± 0.01
Uterus	0.57 ± 0.04^ab^	0.54 ± 0.04^ab^	0.66 ± 0.04^a^	0.45 ± 0.03^b^	0.53 ± 0.03^b^

*Note:* The organ-to-body weight ratio was assessed and compared among the control, DEHP-, DEHP + Vitamin E–, DEHP + Na_2_SeO_3_–, and DEHP + Vitamin E + Na_2_SeO_3_–exposed groups of adult female mice. Values are presented as mean ± SEM (*n* = 3). Statistical significance is indicated by letters (a, b, c).

## Data Availability

The data that support the findings of this study are openly available in https://doi.org/10.5281/zenodo.17290485.
